# Assessment of Vulnerability to Coccidioidomycosis in Arizona and California

**DOI:** 10.3390/ijerph14070680

**Published:** 2017-06-23

**Authors:** Jennifer Shriber, Kathryn C. Conlon, Kaitlin Benedict, Orion Z. McCotter, Jesse E. Bell

**Affiliations:** 1 Rollins School of Public Health, Emory University, Atlanta, GA 30322, USA; jesse@cicsnc.org; 2 Climate and Health Program, Centers for Disease Control and Prevention, Atlanta, GA 30341, USA; kconlon@cdc.gov; 3 Mycotic Diseases Branch, Centers for Disease Control and Prevention, GA 30333, USA; jsy8@cdc.gov (K.B.); yim4@cdc.gov (O.Z.M.); 4 North Carolina Institute for Climate Studies, North Carolina State University, Asheville, NC 28801, USA

**Keywords:** coccidioidomycosis, valley fever, vulnerability, climate change, climate variability, vulnerability index

## Abstract

Coccidioidomycosis is a fungal infection endemic to the southwestern United States, particularly Arizona and California. Its incidence has increased, potentially due in part to the effects of changing climatic variables on fungal growth and spore dissemination. This study aims to quantify the county-level vulnerability to coccidioidomycosis in Arizona and California and to assess the relationships between population vulnerability and climate variability. The variables representing exposure, sensitivity, and adaptive capacity were combined to calculate county level vulnerability indices. Three methods were used: (1) principal components analysis; (2) quartile weighting; and (3) percentile weighting. Two sets of indices, “unsupervised” and “supervised”, were created. Each index was correlated with coccidioidomycosis incidence data from 2000–2014. The supervised percentile index had the highest correlation; it was then correlated with variability measures for temperature, precipitation, and drought. The supervised percentile index was significantly correlated (*p* < 0.05) with coccidioidomycosis incidence in both states. Moderate, positive significant associations (*p* < 0.05) were found between index scores and climate variability when both states were concurrently analyzed and when California was analyzed separately. This research adds to the body of knowledge that could be used to target interventions to vulnerable counties and provides support for the hypothesis that population vulnerability to coccidioidomycosis is associated with climate variability.

## 1. Introduction

Coccidioidomycosis is a fungal infection endemic to the southwestern United States, predominantly Arizona and California [1,2]. Also known as Valley Fever, the infection arises from the inhalation of *Coccidioides immitis* and *Coccidioides posadasii* spores [1]. The inhalation of one spore can result in illness; approximately 40% of infected people experience symptoms that can range from mild (e.g., flu-like) to severe (e.g., community acquired pneumonia, meningitis, and disseminated infections) [3,4,5]. Coccidioidomycosis incidence measures are subject to numerous environmental factors, climate, host population susceptibility, human activity, and case detection and reporting practices. Reported coccidioidomycosis incidence has generally increased in the United States since 1998 [6]. Given the overall increase in coccidioidomycosis incidence, the identification of populations and locations that are vulnerable to increased incidence is vital in order to employ effective public health strategies for this disease.

Risk factors make certain people more susceptible to the severity of coccidioidomycosis infection. Older adults and children under the age of five are at higher risk, and the incidence rates in California among these age groups have increased disproportionately over the past decade [7,8,9]. Older populations are at higher risk of infection as they may have weaker immune systems or concurrent medical conditions that affect their overall health [3,9,10,11,12,13,14]. Younger children are generally more vulnerable than adults to environmental exposures, owing in part to their tendency to breathe more air in proportion to their weight and their differential exposure to dust [15]. A wide range of health conditions including cancer and HIV further increase the risk for severe coccidioidomycosis by compromising the immune system [13,16,17,18]. Immune system suppression may explain the risk of coccidioidomycosis among smokers, which has been observed in Californian and Arizonian populations [12,19,20]. It is estimated that the risk of developing disseminated coccidioidomycosis is 10 to 175 times greater in people of African-American and Filipino decent compared with whites [20]. Host genetic factors have been suggested as an explanation for this racial/ethnic predisposition to severe disease [10,21]. More generally, Cutter et al. describe a variety of factors that contribute to social vulnerability to environmental hazards and therefore could increase risk to diseases such as coccidioidomycosis [22]. These include socio-economic status and educational attainment.

Certain environmental conditions are conducive to *Coccidioides* spp. growth and exposure to spores. Fisher et al. note that sparsely vegetated areas may be more favorable for growth, while cultivated fields and heavily vegetated, paved, or urbanized areas are less ideal [23]. Meanwhile, areas with higher population density could expose more individuals to spores. Edwards and Palmer used skin tests to identify areas in which coccidioidomycosis was endemic, noting localization in the southwestern United States, with the highest prevalence found in counties in California, Arizona, and Texas [24]. However, *Coccidioides’* true geographic distribution is likely broader than previously recognized [25].

Climatic conditions such as precipitation and temperature can impact coccidioidomycosis incidence rates [1,3,4,6,11,13,18,26]. *Coccidioides* spp. require moisture for lifecycle completion. However, when disturbed in the environment, particularly during periods of low precipitation or drought, the spores can become airborne and potentially inhaled [7]. An abundance of moisture can be prohibitive for *Coccidioides* spp.; it is hypothesized that overly moist conditions facilitate the growth of competitors [27]. Several studies noted moderate to high correlations between incidence and antecedent precipitation in Arizona [1,18,28]. Studies performed in California, meanwhile, have found weak correlations between coccidioidomycosis and precipitation [26,29]. Numerous studies found that drought and lower antecedent rainfall were significantly associated with the increased incidence of symptomatic coccidioidomycosis in both Arizona and California [1,3,4,18,28,30,31,32,33]. Further associations have been noted between antecedent temperature and coccidioidomycosis incidence [3,18,28,31]. Reed notes that higher temperatures during the early stages of development may facilitate the removal of competitors by sterilizing the upper portions of the soil while *Coccidioides* conidia remain viable below the surface [27].

The southwestern United States is uniquely affected by climate change and climate variability. Climate change refers to any change in climate over time, while climate variability encompasses ‘variations in the mean state and other statistics (such as standard deviations, the occurrence of extremes, etc.) of the climate on all spatial and temporal scales beyond that of individual weather events’ [34]. Projected future climatic changes for the Southwest include continued warming, decreased precipitation, and more frequent and severe droughts [2]. Many people in the region will be exposed to conditions that may lead to adverse health outcomes as a result of changes in climate, including factors that could lead to the expansion of the range and increase the distribution of *Coccidioides* spp. [35,36,37,38]. The severity of adverse health outcomes depends on an individual or group’s vulnerability to climate-related health impacts. The Intergovernmental Panel on Climate Change’s (IPCC) Fourth Report defines vulnerability as a function of “the character, magnitude, and rate of climate change and variation to which a system is exposed, the sensitivity and adaptive capacity of that system” [39]. These three components, exposure, sensitivity, and adaptive capacity, are crucial to understanding population vulnerability to climate change and its effects on human health [34,40,41]. The most vulnerable groups are those that are most frequently exposed to climate-related hazards, most sensitive to their negative effects, and least resilient to recovery [42].

Identifying populations that are vulnerable to coccidioidomycosis is an important public health challenge. While many studies have assessed vulnerability to environmental hazards and climate change, to our knowledge, there are no published studies that have used vulnerability indices to assess population vulnerability to coccidioidomycosis [22,42,43,44,45,46,47,48,49,50,51,52,53,54,55,56,57]. The purpose of this study is to assess county-level population vulnerability in Arizona and California to high incidence of coccidioidomycosis, with specific consideration given to the role of climate variability in exposure. The specific aims of the study are twofold: (1) to describe counties’ vulnerability to coccidioidomycosis based on indicators representative of exposure, sensitivity, and adaptive capacity; and (2) to examine the association between vulnerability and climate variability in these counties. This study will improve the understanding of vulnerability to coccidioidomycosis in Arizona and California.

## 2. Materials and Methods

### 2.1. Data Collection

Vulnerability was defined using the IPCC’s Fourth Report definition, which gives vulnerability to climate change as the following function [39]:

*Vulnerability* = *Exposure* + *Sensitivity* − *Adaptive Capacity* (1)

Exposure, sensitivity, and adaptive capacity variables were selected based on the literature and availability of data. Table 1 presents an overview of the variables used for the vulnerability index to represent the exposure, sensitivity, and adaptive capacity components. County level demographic variables (e.g., age, race/ethnicity, poverty, education) were obtained from the 2010 U.S. Census [58]. All data were tabulated by county in ArcGIS 10.3.1 (ESRI, Redlands, CA, USA) using TIGER/Line Shapefiles [59,60].

Coccidioidomycosis is currently a reportable disease in 22 states and has been nationally notifiable to the U.S. Centers for Disease Control and Prevention (CDC) through the National Notifiable Diseases Surveillance System (NNDSS) since 1995. County-level NNDSS case counts in Arizona and California from 2000–2014 were used in this analysis [67]. Case counts for some California counties were very sparse and, therefore, excluded.

Land cover raster data were categorized into a binary variable based on criteria noted by Fisher et al., with 0 being unsuitable for *Coccidioides* spp. growth and 1 being suitable for growth [23]. Developed, open space; barren land; shrub/scrub; and grassland/herbaceous categories were assigned a “1”, while all other land types were designated as “0”. The land cover suitability variable represented the percent of raster points in each county with an assignment of “1”. The number of hospitals per 100 square miles was derived from the American Hospital Association and the 2010 geographic area of each county.

Climate data were obtained from the National Oceanographic and Atmospheric Administration (NOAA) National Centers for Environmental Information (NCEI) [68]. Monthly minimum, maximum, and average temperature, precipitation, and drought index (Standardized Precipitation-Evapotranspiration Index, SPEI) were downloaded for all weather stations within California and Arizona for 2000–2014. Normal monthly climate data, including standard deviations, spanning the period of 1981–2010 were also obtained from the NCEI [69].

Climate data were aggregated to the county level, and seasonal and annual minimum, maximum, and average values were computed. Standard deviations for the climate normal data were used to calculate county-level Z scores to indicate by how many standard deviations the seasonal climate indicators for the study period varied from the normal climate.

### 2.2. Descriptive Analysis

Monthly and annual county population estimates from the 2010 U.S. Census were used to calculate coccidioidomycosis incidence rates per 100,000 people for California and Arizona. Linear interpolation was performed to estimate monthly population estimates when those values were missing. Moran’s I and Local Indicators of Spatial Autocorrelation (LISA) statistics were computed to assess clustering of coccidioidomycosis incidence rates among counties. The 2010 incidence rate was included in the analyses as many of the index variables originated from the 2010 U.S. Census. The mean annual and seasonal coccidioidomycosis incidence rates for the study period were used to reduce bias. The descriptive statistics were generated in R (R Foundation for Statistical Computing, Vienna, Austria) [70]. The results were displayed graphically and mapped in order to visually assess any patterns or trends.

### 2.3. Coccidioidomycosis Vulnerability Indices

#### 2.3.1. Methods for Creating Coccidioidomycosis Vulnerability Indices

The variables representing the exposure, sensitivity, and adaptive capacity components were combined to calculate county level vulnerability indices. Three methods were used to calculate the indices: (1) principal components analysis; (2) quartile weighting; and (3) percentile weighting. R was used to run a principle components analysis with varimax rotation; scree plots, eigenvalues, and factor loading were considered when interpreting the resulting principal components. The quartile weighting method assigned values to each variable based on which state-specific quartile they fell into. County-level variables that fell into the first, second, third, or fourth quartile for each state received a weight of 0.25, 0.5, 0.75, or 1, respectively. The mean of these values was calculated to be a components’ total value. Similarly, the percentile weights method used state percentiles to weight each variable, with a maximum weight of 100 for each variable.

Component scores were summed to construct the overall vulnerability score (Equation (1)). Equal weighting was assumed for all components due to a lack of literature supporting differential impacts among exposure, sensitivity, and adaptive capacity. The additive model was selected as, compared to the multiplicative approach, this would not assign a component a score of zero, potentially nullifying other vulnerability components [22,51,71].

Two sets of indices, “unsupervised” and “supervised”, were created using the three methods described above. This was done in consideration of the numerous ways indices can be created, with the specific interest in how sensitive the index outputs are to the methods used [72]. The unsupervised indices included variables identified *a priori* in the literature and assigned them to exposure, sensitivity, and adaptive capacity components. The supervised indices included only those variables that were significantly correlated with coccidioidomycosis incidence. Correlations between the vulnerability index variables and coccidioidomycosis incidence rates were assessed using Spearman rank correlation coefficients. ArcGIS was used to assign composite vulnerability scores for exposure, sensitivity, and adaptive capacity index components for each county.

#### 2.3.2. Validating Coccidioidomycosis Vulnerability Indices

The vulnerability indices were validated by computing Spearman rank correlation coefficients to assess linear associations between the index scores and coccidioidomycosis incidence rates at the county level. Incidence data were not available for some California counties; these locations were excluded from the validation data. Monte Carlo simulation Moran’s I and Anselin Local Moran’s I statistics were computed for the best-performing index to assess the clustering of vulnerability among counties.

### 2.4. Climate Variability & Coccidioidomycosis Vulnerability

Given the short time-scale of this study, climate variability was used as an indicator of broader climate change. The absolute values of the climate Z scores were summed to produce seasonal and overall climate variability scores. These scores indicate any deviations of the 2000–2014 study period values from the baseline normal (1981–2010) temperature, precipitation, and SPEI: higher deviations indicate higher exposures to changes in climate [51]. Spearman rank correlation coefficients were calculated to assess any linear relationships between the vulnerability index score and the climate variability score for each county.

## 3. Results

### 3.1. Descriptive Analysis

**Figure 1:**
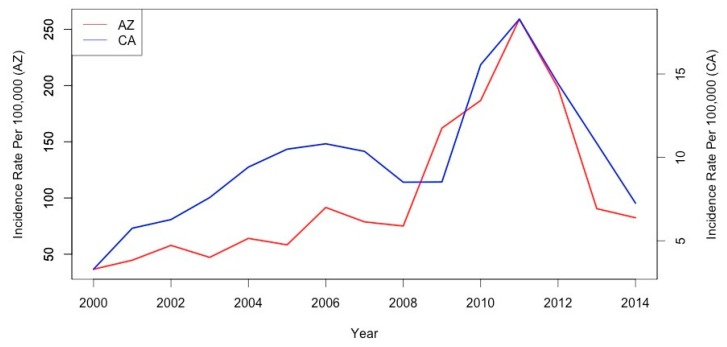
Annual coccidioidomycosis incidence rates for Arizona and select California counties.

Coccidioidomycosis incidence data were analyzed for 20 of California’s 58 counties and all 15 of Arizona’s counties. The coccidioidomycosis incidence rates are presented in Figure 1. Arizona county cases during the study period ranged from 1 to 13,362, with a mean of 430.3 and a median of 21. In California, county cases ranged from one to 2714, with a mean of 140 and a median of 44. Moran’s I indicated no global spatial autocorrelation of county-level incidence rates in Arizona; positive global spatial autocorrelation was present in California, with high incidence rates tending to cluster together throughout the state. In Arizona, local clusters of high incidence rates were located in the southern counties of Maricopa, Pinal, and Pima. Clusters of high seasonal incidence rates occurred in California’s Los Angeles and Orange counties.

The exploratory correlation results are displayed in Table 2. In Arizona, significant positive linear relationships were observed between coccidioidomycosis incidence and the percentage of people living with HIV/AIDS (PLWHA), percent positive skin tests, and the number of hospitals per 100 square miles; no significant negative linear relationships were observed. In California, significant positive relationships were observed between the incidence and the percentage of the population younger than five years, the percentage of the population below the poverty level, the percentage of the population with no higher education, and the percentage of positive skin tests. The percent of the population older than 65 years, the percent of the population of Filipino descent, the cancer incidence rate, the population density, primary care physicians (PCPs) per 100,000 people in the population, and the number of hospitals per 100 square miles had significant negative linear relationships with coccidioidomycosis incidence.

**Figure 2:**
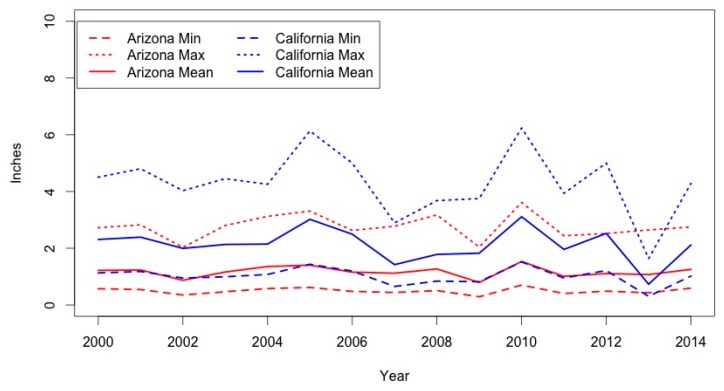
Inter-annual precipitation for Arizona and California.

Significant climate variability is evident among the annual mean, minimum, and maximum precipitation and SPEI in both states (Figure 2). While intra-annual temperature follows the same patterns in both states, with peaks in the summer and low points in December, precipitation and SPEI seasonality differs between the two states. Arizona receives the most precipitation during the late summer months; accordingly, its SPEI also peaks during these months. California, meanwhile, experiences the most rain during the late fall and winter. Intra-annual SPEI values for California reflect this trend.

**Figure 3:**
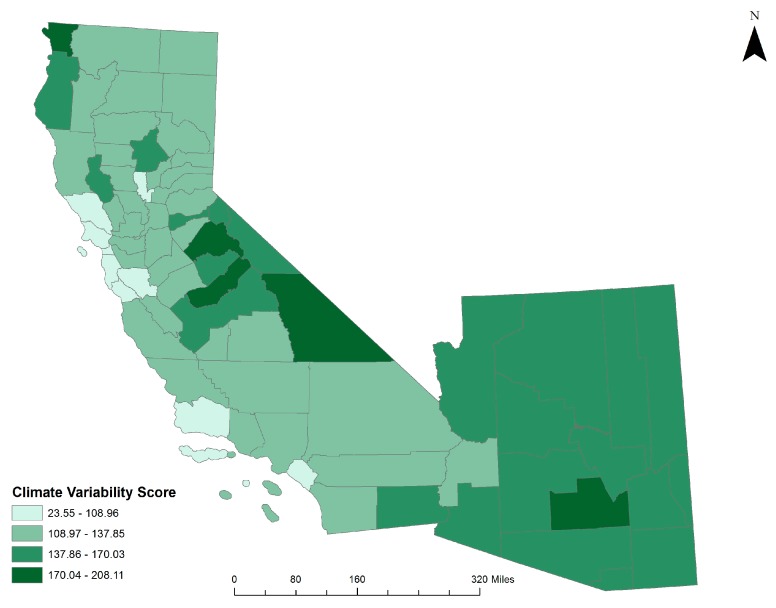
Overall climate variability scores for Arizona and California.

Climate variability scores were calculated individually, seasonally, and cumulatively per climate variable (temperature, precipitation, and SPEI). California’s northern Del Norte County and central Madera and Tuolumne counties experienced the most overall variability from climate normal during 2000–2014, while Pinal County, located in southern Arizona, experienced the most climate variability in that state (Figure 3). Arizona experienced more overall climate variability than California; Arizona counties had more overall seasonal variability, as well as variability in terms of temperature, precipitation, and SPEI throughout the study period. The highest average overall seasonal variability for both states was observed in the fall, while the lowest was in the spring for California and the winter for Arizona. Temperature accounted for the highest amount of variability for both states across the entire study period.

### 3.2. Coccidioidomycosis Vulnerability Indices

A total of four indices, quartile and percentile indices based on both the literature and indicator correlations, were created for each state. The principal component analysis results were not meaningful (e.g., only one factor was created), potentially due to the small number of variables included in the analysis; this method was therefore not used to create additional indices. The validation results are presented in Table 3. The supervised percentile index performed best in all cases. There were significant, positive correlations between this index and all iterations of coccidioidomycosis incidence, although the results varied by state.

The supervised quartile index was also significantly correlated with incidence, though with weaker correlations than the supervised percentile index, while the two unsupervised indices were significantly correlated with incidence for California only.

**Figure 4:**
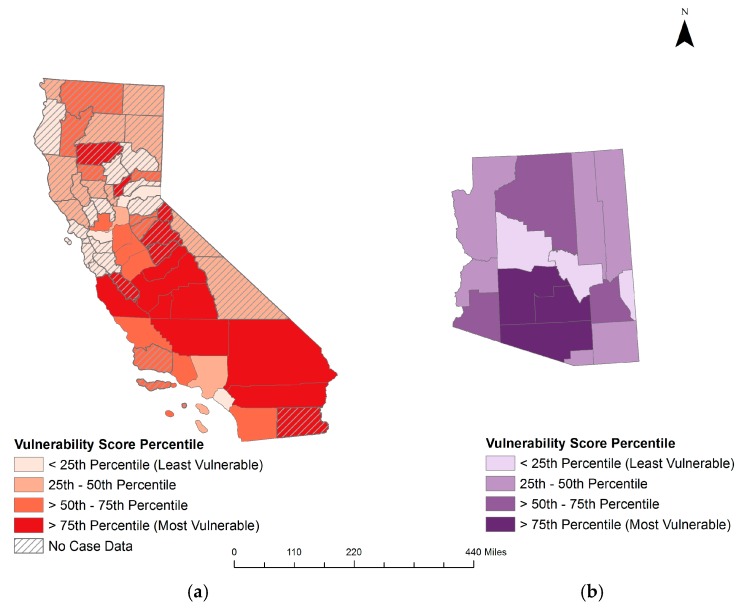
Coccidioidomycosis supervised vulnerability index calculated with state-specific percentile scores for (**a**) California and (**b**) Arizona.

In Arizona, the index scores ranged from 14 (Gila and Yavapai counties) to 107 (Pinal County), with a mean of 47.33 and a standard deviation of 25.54. In California, the scores ranged from −5.5 (Alameda County) to 104.67 (Tulare County), with a mean of 50.5 and a standard deviation of 25.54. Figure 4 presents the index scores based on the percentiles for each state. Based on the indices, the Pinal, Pima, and Maricopa counties, located in the southern region of the state, are most vulnerable to increased coccidioidomycosis incidence in Arizona. In California, San Joaquin Valley’s Tulare, Madera, and Kern counties are the most vulnerable. These counties consistently had some of the highest incidence rates for their respective states during the study period.

The coccidioidomycosis supervised vulnerability index scores were positively globally autocorrelated when considering the two states together and California separately, indicating that uniform patterns are present throughout these geographic areas. Local clusters were present for the two states combined, as well as both Arizona and California separately. Most local autocorrelation was clustering of high vulnerability index scores; in California, this was evident in the San Joaquin Valley and southern counties, while in Arizona clustering was present in the Maricopa, Pima, and Pinal counties. These trends were also evident when Arizona and California were analyzed together. Local clustering of low index scores was present in the counties surrounding San Francisco.

### 3.3. Climate Variability & Coccidioidomycosis Vulnerability

The Spearman rank correlation coefficients assessing correlation between vulnerability scores and climate variability are presented in Table 4. When comparing the vulnerability scores for counties in both states combined, there were significant linear associations with many of the climate variability scores with the exception of several seasonal and all winter variability scores. There was a negative association between all precipitation variability scores and vulnerability index scores. The Arizona index scores were not significantly correlated with any iterations of climate variability. The California index was significantly correlated with most of the climate variability scores. While most of the correlations with precipitation variability scores were negative, the rest of the variability scores were positively correlated with the vulnerability index scores.

## 4. Discussion

This study aimed to describe Arizona and California counties’ vulnerability to coccidioidomycosis based on their exposure, sensitivity, and adaptive capacity and to examine the association between vulnerability and climate variability in these counties. The supervised percentile index was most highly significantly correlated with both coccidioidomycosis incidence and climate variability. While the results were stronger for California than Arizona, this tool is the first for identifying counties whose populations are most at risk of increased coccidioidomycosis incidence based on the IPCC’s definition of vulnerability. It also demonstrates that counties with higher climate variability are more vulnerable.

The initial Spearman rank correlations between the coccidioidomycosis vulnerability index variables and the incidence rates yielded results that were unexpected given the literature on coccidioidomycosis risk factors. While older age, race/ethnicity, socioeconomic factors such as education and poverty levels, and pre-existing medical conditions have been documented to increase the risk of severe coccidioidomycosis, these characteristics had negative correlations with incidence [3,7,9,10,13,16,17,28,73]. This may be due to the small percentage of the population that is made up of people with these characteristics. For instance, Filipinos account for only 0.01–1.60% of the counties in this study population [58]. Even though people of Filipino ethnicity are at a higher risk for severe or disseminated coccidioidomycosis, their scarce numbers may not substantially contribute to the vulnerability of the county as a whole. Additionally, localized changes such as land development or agricultural expansion could engender shifting burdens of disease to populations with different characteristics than those originally presented in the literature. Guevara et al. note that agricultural expansion in parts of Los Angeles County may have led to higher incidence among ethnic populations that are more likely to undertake agricultural work [9].

The highly endemic nature of coccidioidomycosis in Arizona could mask individual risk factors for more severe illness, resulting in few of the vulnerability index variables being significantly correlated with disease incidence. Most of the Arizona population resides in the highly endemic Maricopa, Pima, and Pinal counties, while most California residents live in areas in which *Coccidioides* spp. are not as prevalent [17]. This heightened population exposure causes residents of Arizona to be at risk of infection regardless of their individual characteristics or risk factors. As Arizona’s largest, and presumably most diverse, cities are located in these counties, their demographic and socioeconomic makeup could further mask the true associations between risk factors and coccidioidomycosis incidence at the county level. Linked incidence and patient data or analyses at a finer geographic scale would be ideal to assess the linkages between risk factors from the literature and coccidioidomycosis incidence.

Supervised vulnerability indices represent vulnerability to coccidioidomycosis reasonably well. The supervised percentile vulnerability index was moderately correlated with all iterations of coccidioidomycosis incidence used in the analysis. The index performed best when considering California only. Based on incidences, the index accurately captured high vulnerability in the San Joaquin Valley population, located centrally within the state, as well as the low vulnerability in the San Francisco area and parts of eastern California. The index performed less well in the northern counties; while these counties received high vulnerability index scores, their climate and environment make them ill-suited for *Coccidioides* and they report few to no cases each year [11,17,18,67,74]. The southern Arizona counties of Pinal, Pima, and Maricopa were correctly assigned high vulnerability index scores that matched their high incidence rates.

While Arizona is known to have much higher statewide incidence rates than California, it is interesting to note that the mean and maximum index scores were higher for the state of California [6]. This lack of accord between the vulnerability scores and incidence suggests that other factors play a role in coccidioidomycosis vulnerability. *Coccidioides* spp. require specific habitats and climate conditions to thrive. Actual exposure to *Coccidioides* spores may not occur at the location or even in the county where the growth occurs. It is likely that counties with high vulnerability scores but low incidence rates lack the proper characteristics for fungus growth and spore dissemination. Rural counties that lack these environmental characteristics may also have higher levels of social vulnerability, thereby raising their index scores. Similarly, counties with low vulnerability scores and high incidence may see more cases because they are better suited to *Coccidioides* spp. despite lacking susceptibility factors. Coccidioidomycosis incidence data are based on passive surveillance, which likely underestimates the true number of cases and may reflect differences in county- and state-level case detection and reporting practices. This may be especially true in less-endemic areas where health professionals might be less likely to test patients for coccidioidomycosis [9].

The findings from this study support the hypothesis that climate variability is associated with coccidioidomycosis vulnerability. The California data show significant positive linear relationships between supervised percentile vulnerability index scores and climate variability, particularly in the spring, summer, and fall. The same is demonstrated when considering data from Arizona and California together. The Arizona data did not show a statistically significant linear relationship; this could once again be due to the saturation of coccidioidomycosis in the state that masks underlying trends. While precipitation variability was negatively correlated with index scores, this could be due to the fact that precipitation conditions can both help and hinder *Coccidioides* spp. growth and dissemination. Dry conditions are essential for spore dissemination; however, initial moisture is needed for the fungus to grow according to the ‘grow and blow’ hypothesis [1,11,18,31]. Therefore, variability in the form of too much or too little rain would impede the spread and growth of *Coccidioides* spp. spores, respectively. The results demonstrate that counties with high climate variability, whether in terms of temperature, SPEI, or overall seasonal climate variability, are more vulnerable to coccidioidomycosis incidence. Additional research into the associations between climate and coccidioidomycosis could further clarify this relationship.

### Limitations

Vulnerability indices are inherently limited by their input data. The data available for the coccidioidomycosis vulnerability index was available at the county level. *Coccidioides* spp. are affected by climate and environmental pressures that occur at very fine geographic scales: the index fails to capture such place-specific fluctuations within each county. The generalization of all study data to the county level could mask trends that occur at a smaller scale. An index with greater spatial resolution or granularity that can identify sub-county pockets of vulnerability would prove most useful for identifying populations that are at risk of high coccidioidomycosis incidence rates; however, this may also have high uncertainty due to small numbers.

Supervised indices selected only those variables that showed significant correlation with incidence rates. While this may present a bias towards the correlation of coccidioidomycosis incidence rates, it assures that the creation of the index does not include variables that do not contribute to the overall vulnerability.

The nature of coccidioidomycosis epidemiology made it difficult to assign indicators of exposure. The use of the Edwards and Palmer skin test data as a proxy for the broad environmental distribution of coccidioidomycosis limited the vulnerability index’s effectiveness, as it is now known that *Coccidioides* spp. can live outside the traditionally defined areas of the United States [25]. Nevertheless, this data remains the most comprehensive available despite its age. Further exploration of geographic risk factors would enhance the ability of the vulnerability index to quantify vulnerability to such a geographically restricted disease. While the ‘Percent of Suitable Land’ index variable further attempted to define *Coccidioides* spp.’s ecological niche using readily available data, future research is needed to refine this methodology. Baptista et al. and Lauer et al. have done preliminary research in this area, but mapping *Coccidioides* spp.’s ecological niche both at a fine geographic scale and a broad geographic scope remains a challenge [75,76]. As *Coccidioides* spp. spores can travel long distances once airborne, the presence of suitable conditions in one location does not guarantee higher exposure in that area. Future vulnerability indices would be strengthened by the use of exposure indicators that account for spore movement.

Finally, the mobility of individuals means that some information bias may be present regarding the county of infection. Incidence data fail to capture mobile populations in the two states, particularly migrant workers and older individuals who travel to the area for the winter. Coccidioidomycosis case counts were based on location of residence and may not accurately represent the county in which people were infected, thereby skewing validation results.

## 5. Conclusions

Overall, coccidioidomycosis incidence has increased during the last decade, possibly due in part to changing climate pressures that affect the fungus’ growth and dissemination. We created a vulnerability index for counties in Arizona and California using indicators of exposure, sensitivity, and adaptive capacity. This index displays the counties whose populations are most at risk of increased coccidioidomycosis incidence and demonstrates that counties with high climate variability are more vulnerable. This research adds to the body of knowledge that can be used to target adaptation measures such as public awareness campaigns and to provide training to the most vulnerable counties and groups. The findings can be used to inform vulnerable populations and the public in general of their risks for coccidioidomycosis in order to reduce future morbidity and mortality. Future research is needed better capture coccidioidomycosis vulnerability in Arizona and to display vulnerability at a finer geographic scale for both Arizona and California. This work can also be expanded to investigate the role of future climate variability on changes in vulnerability.

## Figures and Tables

**Table 1:** Characteristics of vulnerability index data.

Variable	Component	Source (Year)
% Land Suitable for *Coccidioides* spp. Growth	Exposure	Multi-Resolution Land Characteristics Consortium (2011) [61]
% Positive Skin Test	Exposure	Edwards & Palmer (1957) [24]
Population Density Per Square Mile	Exposure	U.S. Census (2010) [58]
% Population > 65 years	Sensitivity	U.S. Census (2010) [58]
% Population < 5 years	Sensitivity	U.S. Census (2010) [58]
% Population of African-American Race	Sensitivity	U.S. Census (2010) [58]
% Population of Filipino Race	Sensitivity	U.S. Census (2010) [58]
% Population Below Poverty Level	Sensitivity	U.S. Census (2010) [58]
% Population with No Higher Education	Sensitivity	U.S. Census (2010) [58]
% Population Living with HIV/AIDS	Sensitivity	CDC National Center for HIV/AIDS, Viral Hepatitis, STD, & TB Prevention (2012) [62]
Cancer Incidence Rate Per 100,000 (All Types)	Sensitivity	National Cancer Institute (2012) [63]
% Adults Who Smoke	Sensitivity	CDC Behavioral Risk Factor Surveillance System (2010) [64]
Number of Hospitals Per 100 Square Miles	Adaptive Capacity	American Hospital Association (2012) [65]
Number of Primary Care Physicians per 100,000	Adaptive Capacity	HRSA Areal Health Resource File (2012) [66]

**Table 2:** Spearman rank correlation coefficients for vulnerability index variables and coccidioidomycosis incidence rate (IR).

State	Incidence Rate	% ≥65 Years	% <5 Years	% African American	% Filipino	% Below Poverty Line	Cancer IR	% Adults Who Smoke	% PLWHA	% Adults with No Higher Education	% Suitable Land	% Positive Skin Tests	Population Density Per Sq. Mi.	PCPs Per 100k Population	Hospitals Per 100 Sq. Mi.
Arizona Only	Mean Annual IR	0.12	−0.14	0.46	0.37	−0.40	0.30	0.13	0.49	−0.25	0.21	0.62 *	0.36	−0.06	0.56 *
	2010 IR	0.16	−0.12	0.37	0.32	−0.23	0.16	0.22	0.56 *	−0.18	0.22	0.58 *	0.38	−0.04	0.58 *
	Mean Fall Monthly IR	0.16	−0.26	0.43	0.36	−0.43	0.35	0.14	0.48	−0.35	0.20	0.64 *	0.38	−0.01	0.57 *
	Mean Spring Monthly IR	0.16	−0.18	0.46	0.39	−0.45	0.37	0.12	0.45	−0.25	0.22	0.61 *	0.35	−0.01	0.56 *
	Mean Summer Monthly IR	0.09	−0.13	0.36	0.29	−0.36	0.29	0.15	0.33	−0.16	0.03	0.58 *	0.33	0.07	0.46
	Mean Winter Monthly IR	0.16	−0.18	0.45	0.39	−0.43	0.34	0.11	0.39	−0.17	0.28	0.45	0.22	−0.04	0.44
California Only	Mean Annual IR	−0.47 *	0.65 *	−0.20	−0.46 *	0.66 *	−0.51 *	0.17	−0.27	0.69 *	0.07	0.66 *	−0.65 *	−0.65 *	−0.58 *
	2010 IR	−0.52 *	0.67 *	−0.19	−0.52 *	0.70 *	−0.46 *	0.24	−0.32	0.76 *	0.06	0.63 *	−0.70 *	−0.70 *	−0.62 *
	Mean Fall Monthly IR	−0.49 *	0.66 *	−0.21	−0.51 *	0.69 *	−0.53 *	0.22	−0.30	0.72 *	0.10	0.66 *	−0.71 *	−0.69 *	−0.63 *
	Mean Spring Monthly IR	−0.51 *	0.68 *	−0.17	−0.45 *	0.69 *	−0.50 *	0.18	−0.29	0.69 *	0.08	0.65 *	−0.65 *	−0.63 *	−0.59 *
	Mean Summer Monthly IR	−0.53 *	0.67 *	−0.18	−0.44	0.69 *	−0.49 *	0.20	−0.26	0.72 *	−0.01	0.64 *	−0.64 *	−0.64 *	−0.58 *
	Mean Winter Monthly IR	−0.44	0.64 *	−0.21	−0.47 *	0.63 *	−0.46 *	0.15	−0.29	0.70 *	0.08	0.65 *	−0.66 *	−0.65 *	−0.59 *

* *p* < 0.05.

**Table 3:** Spearman rank correlation coefficients for vulnerability indices and coccidioidomycosis incidence rate (IR).

State	Vulnerability Index Type	Mean Annual IR	2010 IR	Mean Fall Monthly IR	Mean Spring Monthly IR	Mean Summer Monthly IR	Mean Winter Monthly IR
Both States	Quartile Index	0.23	0.24	0.25	0.22	0.17	0.22
	Percentile Index	0.26	0.27	0.28	0.24	0.21	0.25
	Supervised Quartile Index	0.26	0.30	0.29	0.23	0.25	0.23
	Supervised Percentile Index	0.34 *	0.35 *	0.36 *	0.30	0.31	0.31
Arizona Only	Quartile Index	0.22	0.23	0.14	0.18	0.02	0.17
	Percentile Index	0.23	0.26	0.16	0.18	0.05	0.18
	Supervised Quartile Index	0.43	0.50	0.44	0.38	0.38	0.28
	Supervised Percentile Index	0.42	0.43	0.39	0.37	0.32	0.31
California Only	Quartile Index	0.56 *	0.60 *	0.61 *	0.56 *	0.53 *	0.56 *
	Percentile Index	0.62 *	0.66 *	0.66 *	0.60 *	0.60 *	0.62 *
	Supervised Quartile Index	0.63 *	0.68 *	0.66 *	0.61 *	0.62 *	0.64 *
	Supervised Percentile Index	0.72 *	0.77 *	0.75 *	0.71 *	0.72 *	0.72 *

* *p* < 0.05.

**Table 4:** Spearman rank correlation coefficients for supervised percentile vulnerability index scores and climate variability scores.

Climate Variability Score	Both States	Arizona Index	California Index
Overall Variability	0.31 *	0.34	0.42 *
Overall Precip. Variability	−0.23 *	0.08	−0.29 *
Overall Temp. Variability	0.34 *	0.15	0.45 *
Overall SPEI Variability	0.30 *	0.12	0.47 *
Fall Variability	0.21	0.44	0.27 *
Fall Precip. Variability	−0.25 *	−0.15	−0.31 *
Fall Temp. Variability	0.24 *	0.51	0.27 *
Fall SPEI Variability	0.31 *	0.14	0.47 *
Spring Variability	0.15	−0.44	0.29 *
Spring Precip. Variability	−0.25 *	−0.22	−0.26 *
Spring Temp. Variability	0.17	−0.30	0.32 *
Spring SPEI Variability	0.21 *	−0.07	0.37 *
Summer Variability	0.34 *	0.27	0.43 *
Summer Precip. Variability	−0.10	0.09	−0.12
Summer Temp. Variability	0.39 *	0.12	0.45 *
Summer SPEI Variability	0.26 *	0.21	0.40 *
Winter Variability	0.15	0.41	0.10
Winter Precip. Variability	−0.16	0.17	−0.29 *
Winter Temp. Variability	0.21	0.29	0.19
Winter SPEI Variability	0.15	0.26	0.19

* *p* < 0.05.
